# The thrombin generation capability of the Chacma baboon (*Papio ursinus*): implications for haemostatic disease models

**DOI:** 10.1038/s41598-023-50341-8

**Published:** 2023-12-27

**Authors:** J. Joubert, S. M. Meiring, W. J. Janse van Rensburg

**Affiliations:** 1 Department of Haematology and Cell Biology, School of Pathology, Faculty of Health Sciences, University of the Free State, 205 Nelson Mandela Drive, PO Box 339 (G2), Bloemfontein, 9300 South Africa; 2 National Health Laboratory Service, Universitas Academic Laboratories, Haematology, Bloemfontein, South Africa; 3 Human Molecular Biology Unit, School of Biomedical Sciences, Faculty of Health Sciences, University of the Free State, Bloemfontein, South Africa

**Keywords:** Drug development, Experimental models of disease

## Abstract

Baboon models are often used to investigate haemostatic diseases, such as acquired thrombotic thrombocytopenic purpura or bacterial sepsis-induced disseminated intravascular coagulation, and their potential treatment with novel drugs. Thrombin generation is vital for these models, and an important potential therapeutic target. We investigated the thrombin generation profile of the Chacma baboon (*Papio ursinus* – a common pre-clinical model) including the effects of sex and ABO blood group. Thrombin generation curves, lag times, peak heights, times-to-peak, velocity indexes and Endogenous Thrombin Potentials (ETPs) of 40 adult Chacma baboons were assessed and compared with normal human plasma, using a low concentration of tissue factor (1 pM) and phospholipids. Reference intervals were calculated, and results compared between O and non-O ABO blood groups, and between males and females. Lag times of all baboons fell within the human reference interval. Most animals (n = 32; 80%) had times-to-peak above, and velocity indexes and peak heights markedly below (n = 27; 68%) the human range. However, 97.5% of baboons had an ETP above the human reference interval, indicating greater overall thrombin generation. ABO blood group had no effect, but males (n = 14; 35%) had less potent thrombin generation than females (n = 26; 65%), with significantly longer lag times (p = 0.0475), lower peak thrombin concentrations (p = 0.0203), and lower ETPs (p = 0.0238). Chacma baboons have greater overall endogenous thrombin generation potentials than humans, which is even more prominent in females. This should be considered when designing future baboon model experiments involving the haemostatic system, or when evaluating novel therapies in these animals.

## Introduction

The Chacma baboon (*Papio ursinus*) is used frequently as a model of human haemostatic disease and for the evaluation of novel treatment approaches. This includes models of relatively rare conditions such as acquired thrombotic thrombocytopenic purpura (aTTP) which involves the ADAMTS13-Von Willebrand factor-platelet axis^1^, or more common diseases with coagulation-associated abnormalities, such as bacterial sepsis-induced disseminated intravascular coagulation (DIC)^2,3^.

Thrombin generation is a key factor in these models and an important potential therapeutic target. Yet there is no detailed published information available on the thrombin generation capabilities of this species' haemostatic system and, critically, on whether Chacma baboon thrombin generation is at all comparable to that of humans and other non-human primates. Although thrombin generation was assessed as part of a preclinical Chacma baboon study of a synthetic heparin mimetic^4^, thorough characterisation of Chacma baboon thrombin generation was not the primary aim of the study. Similarly, preclinical Olive baboon (*Papio anubis)* studies of protein S^5^ and the factor IXa inhibitor, pegnivacogin, and its reversal agent, anivamersen^6^, as well as a Hamadryas baboon (*Papio hamadryas)* heat-stroke model study of nematode anticoagulant protein (NAP) c2^7^, included some assessment of thrombin generation, but none of these studies primarily aimed to specifically characterise these species’ thrombin generation capabilities in detail. It was, however, explicitly investigated in the Olive baboon, as part of a study comparing thrombin and plasmin generation in humans, Olive baboons, Rhesus monkeys, Yorkshire pigs, Sprague–Dawley rats, New Zealand White rabbits and Hartley guinea pigs^8^, which showed that both Olive baboon and Rhesus macaque thrombin generation are comparable to that of humans.

Whether Chacma baboon thrombin generation is similar to that of other non-human primate species requires investigation and cannot simply be assumed, since interspecies variation has not only been described in both the endogenous thrombin potentials (of humans, rats, pigs and rabbits) and the thrombin generation lag phase times (of humans, rats, pigs, sheep and rabbits), as measured by the thrombin generation assay (TGA)^9^, but also in the Endogenous Thrombin Potentials (ETPs) of different non-human primates, such as Cynomolgus and Rhesus monkeys^10^.

In addition, it is not known whether Chacma baboon thrombin generation is influenced by sex and ABO blood group, as it may be in humans, where elevated thrombin generation potentials have been reported in women^11,12^ and individuals of non-O ABO blood group^13^. This has obvious implications for experimental design.

The aim of this study was to investigate the thrombin generation profile of the Chacma baboon (*Papio ursinus*) and its implications for haemostatic disease models, by determining the thrombin generation curves, lag times, peak heights, times-to-peak, velocity indexes and ETPs of *Papio ursinus* plasma when activated with low concentrations of tissue factor. The effect of ABO blood group and sex on the TGA was also explored.

## Methodology

### Ethics statement

Ethics approval was obtained from the University of the Free State (UFS) Interfaculty Animal Ethics Committee before the commencement of the study (UFS-AED2019/0054). Protocols are evaluated against the South African National Standard for the care and management of laboratory animals (SANS10386:2008). We confirm that all methods were carried out in accordance with relevant guidelines and regulations. All the methods, where applicable, are reported in agreement with the ARRIVE guidelines (https://arriveguidelines.org).

### Experimental animals, sample acquisition and preparation

Importantly, only purpose-bred animals were sampled. No specimens were collected from other animal species. Routine care and phlebotomy were conducted at the Animal Research Centre of the UFS (Bloemfontein, South Africa). Blood was collected from 51 baboons, from which a subgroup of 40 baboons was selected based on their ABO blood groups and sex, to have as broad a representation of the different ABO blood groups and sexes as possible. Only 10 baboons were of non-O ABO blood group and were all included. The rest were all of group O, from which the 30 animals with the largest available number of plasma aliquots (that would be able to accommodate all the planned tests) were selected. Only 14 animals were male and were all included. All 11 baboons not selected for further testing were female and of group O. All 51 animals screened were either adults or young adults.

The exact genetic heterogeneity of the animals is unknown, but it is unlikely that they were all derived from the same original breeding pair (or pairs) since various baboons were historically used for breeding purposes in the colony. The environmental factors were identical for all animals in the colony.

All blood sampling and administration of sedation was performed by a South African Veterinary Council (SAVC) registered veterinarian. Blood sampling was performed under sedation, with ketamine-hydrochloride given intramuscularly every 30 min at 1 mg/kg body weight, as needed. All doses were based on the measured body weight of each animal. Animals' eyelid and toe pinch reflexes were monitored during sedation. Measures to prevent hypothermia during sedation were also followed.

All blood sampling procedures were performed with strict aseptic technique.

Blood specimens were collected into BD Vacutainer® tubes (BD Biosciences, Franklin Lakes, New Jersey, USA) and were drawn from a 20-gauge IV catheter inserted in the femoral/cephalic/great saphenous vein by an experienced veterinarian. A maximum of fifteen separate 5 mL specimen tubes, each containing 0.5 mL 3.2% sodium citrate, were filled with 4.5 mL blood per tube, for a maximum total of 67.5 mL citrated whole blood per baboon. Sampling volumes, therefore, did not exceed 10% of the total blood volume, based on a total blood volume of 70 mL/kg measured body weight^14^. All animals were returned post-sampling to the colony without any adverse events.

Platelet poor plasma was prepared by centrifugation of whole blood at 1500×*g* for 15 min at room temperature, then aliquoted and stored at − 80 °C within 90 min of specimen collection.

### Assays

#### Thrombin generation

Thrombin generation was measured singly on the Technoclone Ceveron Alpha instrument (Technoclone GmbH, Vienna, Austria) using the TECHNOTHROMBIN® TGA kit, according to the manufacturer’s instructions. The TECHNOTHROMBIN® TGA is based on monitoring the fluorescence generated by the cleavage of a fluorogenic substrate by thrombin over time, upon activation of the coagulation cascade by a low concentration (1 picomolar) of recombinant human tissue factor (in Tris-Hepes-NaCl buffer) and negatively charged phospholipids in plasma (RC Low reagent, Technoclone GmbH, Vienna, Austria). From the changes in fluorescence over time, the concentration of thrombin in the sample can be calculated using the reference calibration curve. The increase in thrombin concentration with time then allows calculation of total thrombin generation in the sample. The *Papio ursinus* total endogenous thrombin generation potential and thrombin generation curves were then compared with known data for humans and other animal models.

#### ABO blood group

ABO blood grouping was based on the principle of antibody-induced erythrocyte clumping. Erythrocytes with a given ABO blood group antigen on their surface will agglutinate when exposed to antibodies directed against the given antigen. Briefly, 2–5% erythrocyte suspensions were prepared and mixed with anti-A, anti-B, and anti-AB antibodies to determine the red cell ABO phenotype (forward grouping). Additionally, in the ABO blood group system, as an individual’s serum will naturally contain antibodies directed against the antigen(s) lacking on their erythrocytes, reverse grouping was performed as a confirmatory step, by adding test serum to red cells of known ABO blood group.

### Statistical analysis

Statistical analysis was performed using the EP Evaluator® (version 10.3.0.556, Data Innovations, 2013), Microsoft Excel®, and XLSTAT-Biomed® statistical analysis software systems. Quantitative data were summarised using descriptive statistics, namely frequencies and percentages for categorical data, and means, medians, standard deviations (SDs), coefficients of variation (CVs), and percentiles, for continuous data.

Biological reference intervals were calculated for all relevant parameters, following the Clinical and Laboratory Standards Institute (CLSI) guidelines^15^. The central 95% interval of the distribution, i.e. the interval from the 2.5th percentile to the 97.5th percentile (or upper limit of this interval if appropriate), was defined as the biological reference interval/limit, as stipulated in ISO standard 15,189^16^. The CVs calculated were also compared with published human biological variation values.

To assess whether distribution of the data was normal, D’Agostino-Pearson tests were performed on datasets with repeated values, and Shapiro–Wilk tests on all other datasets.

An unpaired Student’s *t* test was performed to determine if there was a significant difference (p < 0.05) between ABO blood group O and non-O baboons and between male and female baboons for all parameters.

## Results

**Figure 1:**
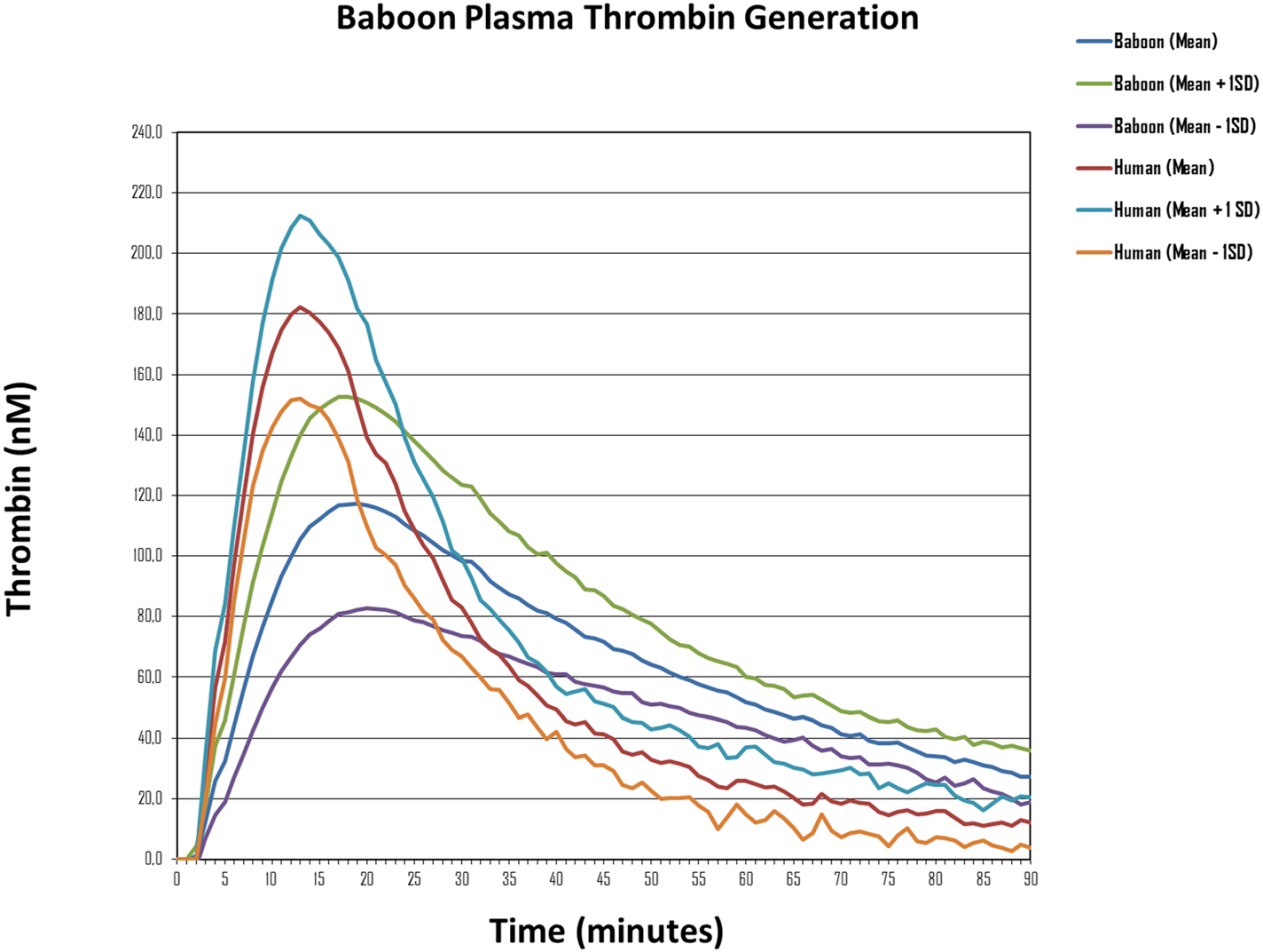
Baboon thrombin generation curve ranges, compared with a selection of normal human plasmas, ran for 90 min. The graph depicts the mean thrombin concentrations of all 40 baboons and 13 normal human plasmas, ± 1SD at the various indicated timepoints. The lower line represents the mean—1SD, and the upper line represents the mean + 1SD. *nM* nanomolar.

The thrombin generation curves of all 40 test animals are shown in Fig. 1 in comparison with curves from a selection of normal human plasmas (n = 13) ran for 90 min. Although there was wide interindividual variation in the thrombin peak, the time-to-peak and lag-time appeared less variable. However, the thrombin generated by baboon plasma did appear to persist for longer than with human plasma. After approximately 30 min, the gradual decrease in the available thrombin was less prominent in the baboon samples than in the human samples, with a protracted residual thrombin “tail” evident from the curves. On an extended run of 120 min (not illustrated in the figure), it was apparent that thrombin concentrations in baboon plasma only reached the 90-min human levels approximately 30 min later, at 120 min.

TGAs also led to the production of a large amount of quantitative data, the statistics of which are summarised in Table 1. All baboon data had a normal distribution, confirmed with either a Shapiro–Wilk or D’Agostino-Pearson normality test.

**Table 1:** The range, median, mean, standard deviation (SD), coefficient of variation (CV), and central 95% interval of the thrombin generation lag time, peak height (maximum thrombin), time-to-peak (time to maximum thrombin), velocity index and endogenous thrombin potential (ETP; area under the curve) of Chacma baboons (n = 40) compared with humans (n = 44).

Parameter	Range		Median		Mean		SD		CV%		Central 95% interval	
	Chacma baboons	Humans	Chacma baboons	Humans	Chacma baboons	Humans	Chacma baboons	Humans	Chacma baboons	Humans	Chacma baboons	Humans
Lag-time (minutes)	3.0–5.0	2.1–8.8	4.0	3.9	4.2	4.0	0.5	1.1	11.4	28.0	4.0–5.0	2.3–6.0
Peak-height (nM)	46.7–198.6	135.9–829.2	114.2	318.7	120.0	348.5	35.7	156.6	29.8	44.9	70.5–184.5	139.6–707.5
Time-to-peak (minutes)	10.0–20.0	4.3–13.3	15.0	7.8	14.8	8.1	2.5	2.0	16.6	24.7	11.0–19.0	5.0–12.7
Velocity index (nM/minute)	4.2–24.8	22.7–284.7	11.3	78.2	12.1	100.9	5.2	69.7	42.6	69.1	5.9–23.1	24.6–270.7
ETP (nM.minute)	3209.4–7850.7	1451.6–5033.3	5687.4	2785.6	5942.0	2770.2	1114.6	602.4	18.8	21.7	4252.7–7796.8	1697.7–3845.7

**Figure 2:**
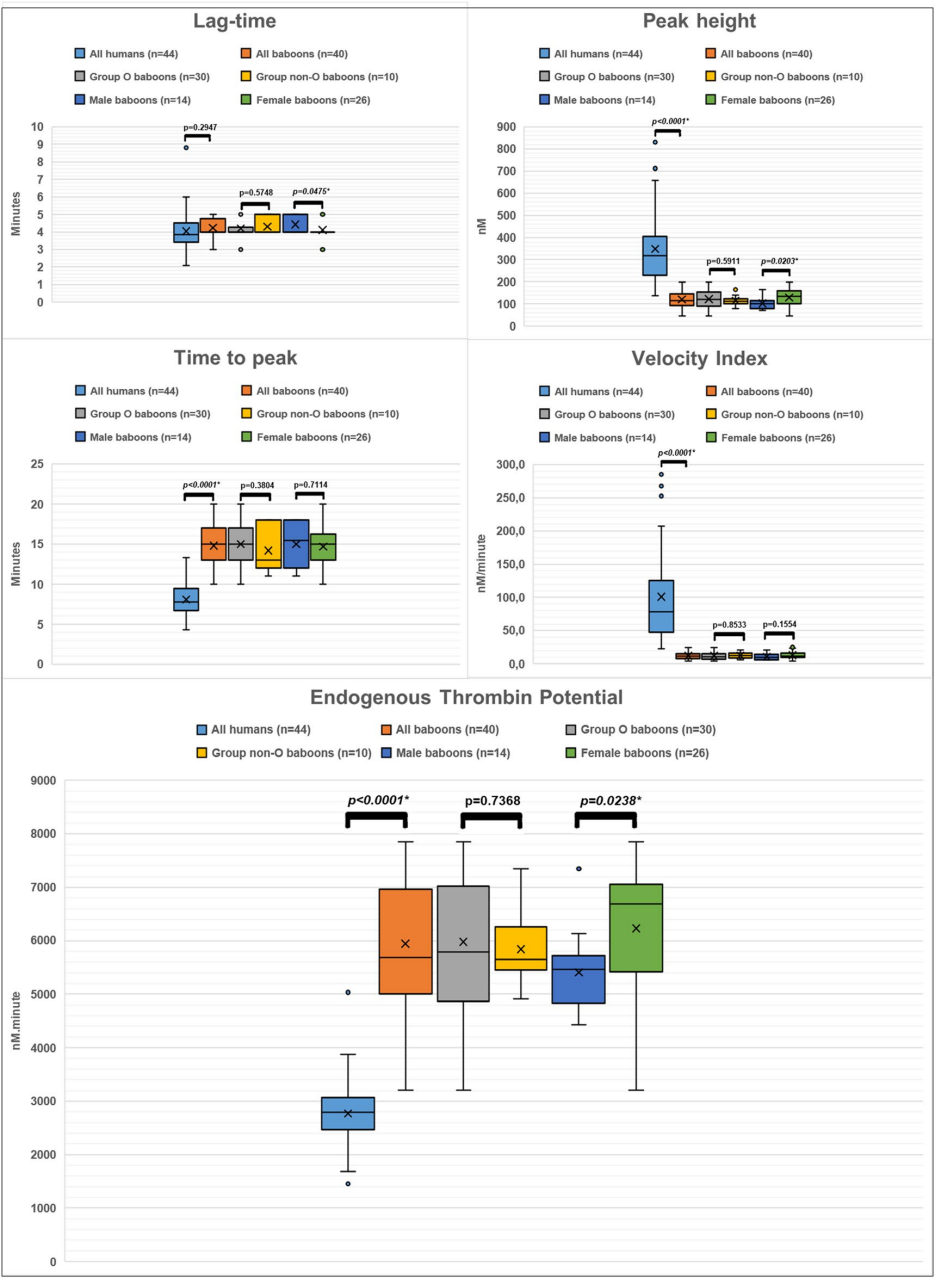
Comparison of *Papio ursinus* thrombin generation parameters of all baboons (n = 40), and all subgroups, with corresponding human data (n = 44).

The baboon data were compared with a normal human reference interval previously derived locally, using the exact same method as outlined above, on plasma aliquots of 44 healthy individuals. These data are also shown in Table 1 and Fig. 2. The human reference interval was also taken as the central 95% interval of the distribution, i.e. the interval from the 2.5th percentile to the 97.5th percentile, as outlined above for the baboon specimens. As there is much variation in how TGAs are performed internationally, it is recommended that laboratories establish and validate their own in-house reference intervals^13^. The local human reference ranges were also previously established using runs of 90 min.

The lag-times for all baboons fell within the human reference range. However, the peak-height values tended toward the lower limit and 27 baboons (68%) had peak thrombin concentrations below the human reference interval. They also had a much narrower range than would be locally accepted as normal in humans (114.0 nM vs 567.9 nM in humans). The majority (n = 32; 80%) of test animals had times-to-peak above the upper limit of the human reference range. It resulted in velocity index values markedly below the human reference range, with only one baboon (2.5%) falling above the lower reference limit. All but one of the baboons tested (97.5%) had an ETP above the human reference range. Therefore, the calculated central 95% reference interval was much higher than the human reference range, indicating greater overall thrombin generation.

### ABO blood group results and influence on assay parameters

Most animals (n = 30; 75%) were ABO blood group O, while the remainder (n = 10; 25%) typed as subgroups of A. No baboons typed as group B or AB. The effects of ABO blood group on the various quantitative and qualitative parameters are summarised in Table 2. There were no significant differences (p < 0.05) found between the different blood groups for any of the parameters measured.

**Table 2:** The effects of ABO blood group on the quantitative parameters of thrombin generation.

Parameter, with human reference range	Group O (n = 30)				Subgroups of A (n = 10)				p-value
	Range	Median	Mean	SD	Range	Median	Mean	SD	
Lag-time, 2.3–6.0 min	3.0–5.0	4.0	4.2	0.5	4.0–5.0	4.0	4.3	0.5	0.5748
Peak-height, 139.6–707.5 nM	46.7–198.6	119.9	121.8	39.2	80–164.2	111.2	114.6	23.2	0.5911
Time-to-peak, 5.0–12.7 min	10.0.20.0	15.0	15.0	2.3	11.0–18.0	13.0	14.2	2.8	0.3804
Velocity index, 24.6–270.7 nM/minute	4.2–24.8	10.8	12.0	5.5	6.2–20.5	12.3	12.4	4.3	0.8533
ETP, 1697.7–3845.7 nM.minute	3209–7851	5785.7	5976.8	1230.5	4908–7344	5644.5	5837.5	699.3	0.7368

### Influence of animals’ sex

Most animals were female (n = 26; 65%, males: n = 14; 35%) and the effects of animals’ sex on the various quantitative parameters of thrombin generation are summarised in Table 3. Males had less potent thrombin generation, evidenced by longer lag times (p = 0.0475), lower peak thrombin concentrations (p = 0.0203), and lower ETPs (p = 0.0238).

**Table 3:** The effects of baboons’ sex on the quantitative parameters of thrombin generation.

Parameter, with human reference range	Females (n = 26)				Males (n = 14)				p-value
	Range	Median	Mean	SD	Range	Median	Mean	SD	
Lag-time, 2.3–6.0 min	3.0–5.0	4.0	4.1	0.4	4.0–5.0	4.0	4.4	0.5	0.0475*
Peak-height, 139.6–707.5 nM	46.7–198.6	135.3	129.5	37.1	71.1–164.2	102.0	102.4	25.8	0.0203*
Time-to-peak, 5.0–12.7 min	10.0–20.0	15.0	14.7	2.4	11.0–18.0	15.5	15.0	2.7	0.7114
Velocity index, 24.6–270.7 nM/minute	4.2–24.8	11.8	13.0	5.3	5.9–20.5	9.7	10.5	4.7	0.1554
ETP, 1697.7–3845.7 nM.minute	3209–7851	6687.2	6230.2	1180.1	4429–7344	5466.5	5406.8	758.1	0.0238*

*Indicates a significant difference (p < 0.05).

Significant values are in bold.

## Discussion

Thrombin generation lag times of all baboons fell within human reference ranges, but most animals (n = 32; 80%) had times-to-peak above (and thus velocity indexes markedly below) the human reference range. However, despite lower peak thrombin concentrations in the majority of test animals (n = 27; 68%) 97.5% of baboons had an ETP above the human reference range (1697.7–3845.7 nM.minute), with a mean ETP of 5942.0 nM.minute (Table 1). This indicates a greater overall thrombin generation potential than humans, primarily due to the effect of a prominent thrombin generation “tail” evident in Fig. 1. This should be considered when designing future experiments involving the haemostatic system or evaluating novel therapies in these animals. Possible explanations for this observation include a less potent natural anticoagulant system in the Chacma baboon than in humans, with lower levels and/or activities of tissue factor pathway inhibitor (TFPI), antithrombin, protein C, protein S, protein Z, protein Z-dependent protease inhibitor, or soluble thrombomodulin, or alternatively, higher factor VIII (FVIII) levels, since the most important determinants of the overall amount of thrombin generated in humans (as quantified by ETP) are FVIII, antithrombin, and free protein S^12,13^. Since our study was conceived as a precursory exploration of Chacma baboon thrombin generation, these factors were not tested in the current study, but will be the subject of future research at our institution.

In humans, the procoagulant effect of FVIII appears to be particularly important when thrombin generation is initiated by low tissue factor concentrations^13^, such as was used in our experiments. Baseline FVIII levels were determined in 15 male Chacma baboons by Jacquemin et al. when investigating the potential clinical utility of the human monoclonal antibody Mab-LE2E9Q (a partial FVIII inhibitor) and reported to generally be in excess of 200 ng/mL^17^. Since this is above the human reference interval of 100–200 ng/mL^18^, higher FVIII levels in the Chacma baboon may explain (at least partially) their greater thrombin generation capability and should be explored further. The role of the natural anticoagulant system, apart from perhaps thrombomodulin which was assessed at baseline by Redl et al.^19^ in a Chacma baboon sepsis model^19^, has not been comprehensively explored in this species yet, creating an opportunity for future research.

Although TGA methods similar to ours have been used in a preclinical Chacma baboon study of a synthetic heparin mimetic^4^ and in preclinical Olive baboon (*Papio anubis)* studies of protein S^5^ and the factor IXa inhibitor pegnivacogin and its reversal agent anivamersen^6^, as well as in a *Papio hamadryas* heat-stroke model study of NAP c2^7^, the overall Chacma baboon thrombin generation potential has not been investigated in detail yet, using a formal TGA. It has, however, been investigated in the Olive baboon, using a simultaneous thrombin plasmin generation assay^20^, as part of a study comparing thrombin and plasmin generation in humans, Olive baboons, Rhesus monkeys, Yorkshire pigs, Sprague–Dawley rats, New Zealand White rabbits and Hartley guinea pigs^8^, which confirmed that the thrombin generation profiles of Olive baboon and Rhesus macaque plasma are broadly similar to that of humans, a finding which mirrored the baseline thrombin generation data of the experiments on pegnivacogin by Bel et al.^6^.

Interestingly, the mean peak thrombin concentrations obtained for Olive baboon (n = 10) and human (n = 28) plasma by Tarandovskiy et al.^8^ is almost four times greater than we obtained for Chacma baboons, and the production rate is approximately ten times higher, although this study used a higher concentration of tissue factor (4.5 pM vs 1 pM in our study) which could explain the observed differences. However, although ETP values are not reported in this study, it is noteworthy that our mean Chacma baboon ETP was well above the human reference range, despite low peak thrombin concentrations and production rates, again demonstrating the profound effect of the persistent thrombin generation tail on total ETP in Chacma baboons. Moreover, this effect was observed despite the low tissue factor concentration (1 pM) used in our experiments. Whether results obtained in the Olive baboon can be directly compared to our Chacma baboon data remains to be explored, but should not be automatically assumed, due to probable interspecies differences already alluded to. In addition, it has been convincingly shown that animal plasmas have widely variable sensitivities to human tissue factor^21^, further complicating interspecies comparison, and human extrapolation, of any TGA results.

Regardless of the differences outlined above, our Chacma baboon ETPs are still more similar to human ETPs than pig, rabbit^9^, Sprague–Dawley rat, and Cynomolgus monkey (*Macaca fascicularis*)^10^, ETPs are. Siller-Matula et al.^9^, after performing TGAs on six members of each species using a similar method to ours, report median ETPs of 2043 nM.minute on pigs and 6295 nM.minute on rabbits, which represents differences of − 52% and + 49%, respectively, from the corresponding human median of 4235 nM.minute. In contrast, our Chacma baboon median ETP of 5687.4 nM.minute is only 34% higher than their human median.

Poitout-Belissent et al.^10^ investigated compound-related effects on coagulability in Sprague–Dawley rats, and Cynomolgus and Rhesus monkeys, using a TGA method similar to ours, and report baseline mean values for the rats and rhesus monkeys used in their experiments, which can be used for indirect comparison. The mean ETP of 664.3 nM.minute reported for rats is 84% lower than the median ETP reported for humans by Siller-Matula et al.^9^, who reported a much higher median ETP for the same rat species (3075 nM.minute, which represents a difference of only − 27% from the human median), highlighting the large interindividual variation possibly present in rats, which may also complicate the use of this species in coagulation experiments. Although mean baseline values for Cynomolgus monkeys are not reported, Poitout-Belissent et al.^10^ do provide reference ranges for various TGA parameters for this species, which they calculated to investigate intra-and interassay variation. Cynomolgus monkey ETPs ranged from 1072.5 to 1827.5 nM.minute (n = 13). The upper limit of this range is still 57% lower than the median human ETP reported by Siller-Matula et al.^9^, indicating that the Cynomolgus monkey is also not the ideal species to use as a model of human thrombin generation. Rhesus monkeys, however, had a mean baseline ETP of 2782.2 nM.minute, which, similar to our Chacma baboon ETPs, represents a difference of − 34% from the reported median human ETP, indicating that this species may also be a suitable model. With a median ETP of 4092 nM.minute, which only differs by − 3% from the median human ETP, sheep are also a possibly suitable species for coagulation studies^9^.

Although formal TGAs were not performed in any of the preclinical studies conducted in the Chacma baboon model of aTTP^1,22–25^, nor in any of the recent DIC/sepsis model experiments in this species^2,3,26,27^, it has implications for these models, as well as for the potential investigation of thrombolytic drugs in the aTTP model. Firstly, since thrombin has also been shown to cleave platelet-bound VWF under flow so that excessive and sustained generation of thrombin would restrict the presence of VWF to its release point^28^, a greater overall ETP may explain why (in contrast to clinical experience with TTP in humans) no animal has ever demised or suffered from excessive bleeding, in any of the preclinical studies conducted in this model. Chacma baboons’ apparently greater (longer) thrombin generation ability may have an ameliorative effect in the aTTP model, with thrombin acting as a backup mechanism for ADAMTS13.

Secondly, a greater ETP could possibly lead to increased activation of TAFI with concomitant greater inhibition of fibrinolysis through TAFI’s removal of C-terminal lysine residues from fibrin, thereby decreasing the capacity of plasminogen and tissue-type Plasminogen Activator (tPA) to bind to fibrin surfaces^29,30^. The resultant indirect inhibition of fibrin breakdown could not only lessen TTP-associated bleeding in this model but also redirect the actions of plasminogen and tPA away from fibrin clots, allowing more plasminogen and tPA to be available for platelet-VWF binding and degradation than would be expected in humans. This implies that should tPA be considered in a future experiment in this model as was recently suggested^25^, less fibrin binding may occur than would be expected in humans, with the presence of more free tPA for non-fibrin specific effects, such as VWF degradation via plasminogen.

Conversely, since increased thrombin generation has been reported as a consequence of thrombolytic therapy with tPA or urokinase-type Plasminogen Activator (uPA) in humans^31,32^, implicating a role for the fibrinolytic system in augmenting coagulation, the Chacma baboon’s greater overall thrombin generation potential should be considered when designing future in vivo preclinical studies of thrombolytic drugs in the aTTP model. The use of therapeutic tPA in the Chacma baboon aTTP model may result in significantly greater thrombin generation than expected from a comparable dose in humans. Thirdly, since excess thrombin generation is central to the pathogenesis of DIC^33,34^, Chacma baboons’ more potent overall thrombin generation ability may influence DIC/sepsis models in this species, potentially leading to more severe DIC phenotypes than would be expected after a similar insult in humans.

Although thrombin generation appears to be increased in humans of non-O ABO blood group^13^, this was not observed in the Chacma baboon. Individual animals’ sex did however influence Chacma baboon thrombin generation capability. Most animals were female (n = 26; 65%, males: n = 14; 35%) but males overall had less potent thrombin generation, with longer lag times (p = 0.0475), lower peak thrombin concentrations (p = 0.0203), and, notably, lower ETPs (p = 0.0238). This is consistent with observations in humans, where thrombin generation was also reported to be increased in women^11–13^. Whether this is due to sex-related differences in FVIII, antithrombin, or free protein S, as is reported for humans^12,13^, remains to be elucidated.

The interindividual variation of Chacma baboons and humans, represented by the CV, is compared in Table 4 with a selection of published human interindividual (between-subject; CV_g_) biological variation values, where available. All parameters of the thrombin generation capability of the Chacma baboon displayed interindividual variation comparable to that of humans. This is encouraging in terms of the continued suitability of this species as an animal model of human haemostatic disease and the translatability of results. Thrombin generation had interindividual variation remarkably similar to that of humans, although the differences outlined in other sections above need to be considered when interpreting results, such as the markedly different reference interval for ETP, for instance.

**Table 4:** Consolidated reference intervals and interindividual variation in the Chacma baboon (n = 40), of the various quantitative parameters of thrombin generation, compared with the local human reference intervals and interindividual variation (n = 44), as well as published human interindividual biological variation, where available.

Parameter	Chacma Baboon reference interval (2.5–97.5%)	Human reference interval for assay	Chacma baboon %CV	Human, %CV	Human %CV_g_, with reference
Lag-time (minutes)	4.0–5.0	2.3–6.0	11.4	28.0	13.9^13^
					14.7^35^
Peak-height (nM)	70.5–184.5	139.6–707.5	29.8	44.9	27.3^13^
					38.0^35^
Time-to-peak (minutes)	11.0–19.0	5.0–12.7	16.6	24.7	14.3^13^
					56.1^35^
Velocity index (nM/minute)	5.9–23.1	24.6–270.7	42.6	69.2	48.2^13^
ETP (nM.minute)	4252.7–7796.8	1697.7–3845.7	18.8	21.8	16.4^13^
					34.3^35^

*%CV* coefficient of variation, *%CVg* interindividual (between-subject) biological variation.

## Conclusion

The thrombin generation capability of *Papio ursinus* plasma outperforms that of human plasma in terms of the overall ETP of especially female Chacma baboons, but still appears to be closer to human values than those of many other commonly used laboratory animal species are. This greater thrombin generation potential may have an ameliorative effect in the aTTP model but can also theoretically lead to more profound abnormalities in DIC/sepsis models than would be encountered in humans under similar conditions. Considering their less potent thrombin generation (which may have an ameliorative effect in TTP) male baboons may potentially be better suited for use in the aTTP model.

## Future studies

Considering their greater thrombin generation potentials, a complete characterisation of the Chacma baboon’s natural anticoagulant system is indicated, which would include estimations of *Papio ursinus* antithrombin, protein C, protein S, protein Z, protein Z-dependent protease inhibitor, soluble thrombomodulin, and TFPI activities and concentrations. Establishing reference intervals for coagulation factor activities, especially for FVIII, will also clarify whether the elevated ETP of *Papio ursinus* is a consequence of a more prominent procoagulant drive or of a less potent natural anticoagulant system with concomitant lower thrombin inhibition.

## Limitations

The main limitation of this study is that the TGA used to characterise Chacma baboon thrombin generation capability, was designed, developed, and intended for use in humans e.g., the fluorogenic substrate was developed to bind human thrombin and not necessarily Chacma baboon thrombin, and the reactions were initiated with human recombinant tissue factor, not baboon tissue factor. Although this is an inherent limitation of most (if not all) animal models, it still limits the accuracy with which actual Chacma baboon haemostasis can be assessed. However, conversely, using human TGAs in this species can also be considered mandatory for two reasons. Firstly, since these are the assays that would ultimately be used in humans to assess the potential effects of any novel treatment, it would be prudent to use these very same assays already during the preclinical testing phase, so that assay variability is at least excluded when data are analysed and extrapolated for later human application. Secondly, since these are accepted, commonly used, generally widely available assays, the use of esoteric, species-specific assays may not only be detrimental to the translatability of any results but may also be impractical due to their possibly limited application outside of the Chacma baboon.

The effects of preanalytical variables are particularly important when investigating thrombin generation^36^ and cannot be completely discounted as potential limiting factors in our study, especially since TGAs were not performed in duplicate, due to cost constraints. Since multiple specimens had to be collected from each baboon simultaneously during a single event, an IV catheter had to be used, which renders the specimens susceptible to haemolysis. However, none of the specimens had any features of haemolysis on visual inspection, and since intravenous catheters are acceptable, provided a proper phlebotomy technique is used and specimens are scrutinised for haemolysis^36^ the potential effect of this on our results is likely negligible. Whole blood specimens were single-centrifuged prior to plasma aliquoting and storage, in order to align with the methods employed in other animal model research^8^, so that results could be more directly compared. Significant differences in ETP have however been described when specimens collected in Monovette® plastic tubes were subjected to single centrifugation vs. double centrifugation^36^. With Vacutainer® tubes (as used in our study), these differences in ETP are reported to be considerably smaller (specifically, 4.7%)^36^, so that the effect of single centrifugation on our results is also likely negligible.

No correction was made for residual α2-macroglobulin-thrombin complex (α2M-T) amidolytic activity. α2M-T is physiologically unimportant but can still convert the fluorogenic substrate and may therefore influence TGA results^37^. However, since our human TGA data were not subjected to this correction either, meaningful comparisons between our human and baboon data can likely still be made.

Even though this is the largest number of individual Chacma baboons ever included in a study involving thrombin generation, the study is still limited by the low number of animals tested. Nevertheless, since a larger number of participants is simply not feasible, the reference intervals calculated from the current data now give researchers a much better idea of the broader context of their quantitative TGA results in this species. Similarly, the low number of males and animals of non-O ABO blood group included in the study is an (unexpected) limitation. Perhaps more pertinently, this observation (reinforced by the complete lack of ABO group B or AB baboons) also implies limited genetic diversity of the UFS breeding colony, which can in itself be seen as a significant limitation of this study. However, it can be argued that this limitation is of no practical importance since these are the actual animals used for experimentation in this unit.

## Data Availability

The datasets used and/or analysed during the current study are available from the corresponding author on reasonable request.
